# Association of long-term exposure to air pollution with sleep among middle-aged and older adults in China: A nationwide study from 2015 to 2018

**DOI:** 10.1371/journal.pone.0336665

**Published:** 2026-03-27

**Authors:** Jing Cao, Zirong Li, Yufei Wu, Tian Ni, Jiwei Zhang, Yuying Xu, Zhengchuan Zhu, Miaoran Wang, Qiuyan Li

**Affiliations:** 1 Beijing University of Chinese Medicine, Beijing, China; 2 Xiyuan Hospital, China Academy of Chinese Medical Sciences, Beijing, China; Korea University - Seoul Campus: Korea University, KOREA, REPUBLIC OF

## Abstract

**Background:**

This study aims to evaluate the associations of long-term exposure to five major air pollutants (PM_2.5_, PM_10_, NO_2_, SO_2_, and CO) with sleep duration and quality among middle-aged and older Chinese adults.

**Methods:**

This study used data from 14856 adults aged 45 and above participating in the China Health and Retirement Longitudinal Study (CHARLS). Changes in nocturnal sleep duration between baseline (2015) and follow-up (2018) were categorized into three groups: reductions ≥1 h, 1.5 h, or 2 h. The increase in the number of days with restless sleep at follow-up was defined as a deterioration in sleep quality. We used the STET model to estimate air pollution exposure and calculate the concentration differences over 1- or 2-year periods preceding each interview. We investigated the associations of exposure differences to five pollutants with sleep duration and quality through generalized linear mixed and ordinal logistic regression models. Interaction analyses were employed to identify potential effect modifiers.

**Results:**

All 1-year exposure differences to air pollution positively correlated with reductions in sleep duration of both ≥1.5 h and ≥2 h. CO exposure demonstrated the highest risk for a ≥ 1.5 h reduction (OR = 1.451; 95% CI: 1.065–1.975) and a ≥ 2 h reduction (OR = 1.557; 95% CI: 1.135–2.135) per 1 μg/m^3^ increment. Higher exposure levels to PM_2.5_, PM_10,_ NO_2_ and SO_2_ correlated with elevated risks of sleep quality deterioration. NO_2_ exposure demonstrated the highest risk, with a 22.3% higher risk per 10 μg/m^3^ increment (95% CI: 1.039–1.439). Pollutants affected sleep duration and quality in varying temporal patterns. Overall, 1-year exposure difference considerably impacted the sleep duration, while 2-year exposure difference significantly impacted the sleep quality. We also found that the air pollution’s adverse impacts on sleep quality were especially significant for those living in urban areas.

**Conclusion:**

Long-term exposure to air pollution is associated with adverse sleep outcomes in middle-aged and older populations.

## Introduction

Sleep is an essential physiological mechanism that supports both physiological and psychological well-being. Good sleep quality facilitates the preservation of metabolic homeostasis, enhances memory, and improves immune function, as well as alleviates emotional states such as anxiety, stress, and depression [1–5]. As age advances, seniors may suffer lower sleep efficiency, shorter total sleep time, more frequent nighttime awakenings, and sleep fragmentation [6]. The Annual Sleep Report of China 2025 reveals that the average sleep time for adults is 7 hours, with 48.5% experiencing sleep problems. This prevalence gradually increases with age. Research has shown that those suffering from sleep disturbances are more prone to experience cognitive decline or even dementia, mental health problems such as anxiety and depression, cardiovascular diseases and even death in middle-aged and older people, severely jeopardizing their quality of life [7–10]. Genetic susceptibility and unhealthy lifestyles such as tea drinking, daytime sleepiness, digital device addiction, and heavy life-work pressures contribute to sleep disturbances [11–13]. However, these traditional factors cannot entirely explain the causes of the increased prevalence of sleep disorders.

Air pollution ranks among the foremost public health threats facing humanity today [14–15], characterized by its complex components, especially fine particulate matter (particulate matter with an aerodynamic diameter less than 2.5 micrometers, PM_2.5_), inhalable particulate matter (particulate matter with an aerodynamic diameter less than 10 micrometers, PM_10_), and gaseous elements such as nitrogen dioxide (NO_2_), sulfur dioxide (SO_2_), and carbon monoxide (CO). Air pollution poses significant threats to respiratory and cardiovascular health. Accumulating epidemiological evidence has further established robust associations between such exposure and sleep disorders [16–19]. Several large-scale epidemiological studies demonstrate that atmospheric pollutants can disrupt sleep architecture via direct or indirect mechanisms and indicate a dose-response relationship between pollutants and sleep disturbances [20–21]. An investigation based on data from 1.2 million nights of wearable devices by a team from Peking University First Hospital found that long-term exposure to ozone (O_3_), NO_2_, SO_2_, CO, and particulate matter (PM_2.5_, PM_10_) resulted in shorter deep sleep duration [20]. A study based on Chinese social media data confirmed the connection between air pollution and sleep deprivation, noting that each standard deviation (SD) rise in air quality index (AQI) corresponded to an 11.6% increase in insomnia and for PM_2.5_, the number was 12.8% [21].

These findings are supported through diverse biological mechanisms such as respiratory dysfunction and central nervous system (CNS) impairment induced by air pollution [22–23], though precise mechanisms underlying pollution-mediated sleep disruption remain incompletely understood. Approximately 94% of people worldwide face PM_2.5_ concentrations exceeding safety standards [24]. Resultant sleep disturbances potentially contribute to long-term health consequences such as neurodegenerative diseases and metabolic syndrome. This is especially relevant in China, given its severe air pollution burden [25]. The health effects of air pollution are well documented; however, evidence for its direct association with sleep disorders in older adults in China is sparse, especially for SO_2_ and CO.

Therefore, within the context of rapid population aging in China, it is crucial to systematically evaluate the associations between sleep and a range of air pollutants, including SO_2_ and CO, to address the current gap in the literature. Utilizing data from a nationwide longitudinal survey, this study aims to assess the associations of long-term exposure to five major air pollutants (PM_2.5_, PM_10_, NO_2_, SO_2_, and CO) with sleep duration and quality among middle-aged and older Chinese adults, and to determine whether the strength of these associations varies by pollutant type.

## Materials and methods

### Study population

Conducted across 450 communities and villages in 150 counties of 28 Chinese provinces, the China Health and Retirement Longitudinal Study (CHARLS) prospectively surveyed middle-aged and older adults. From 2011 to 2018, four waves of investigations were conducted utilizing multistage probability sampling. CHARLS contains demographic information, lifestyle habits, disease conditions, and living environment (urban or rural) of participants.

**Fig 1:**
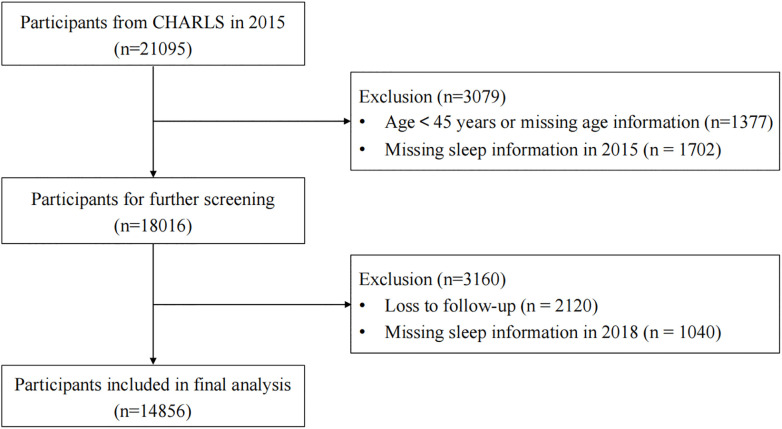
Screening flowchart.

This study utilized data from CHARLS’s 2015 and 2018 surveys. We included participants who had complete sleep data available in both waves. The exclusion criteria were as follows: 1) age < 45 years or missing age information in 2015; 2) missing sleep information in 2015; and 3) loss to follow-up or missing sleep information in 2018. Following these criteria, a total of 14856 participants were retained for the final analysis. The detailed participant selection process is shown in Fig 1.

Ethical approval for the CHARLS protocol was granted by the Ethics Review Board of Peking University (No. IRB00001052–11015), and all participants provided written informed consent. Our current work constitutes a secondary analysis of completely de-identified, publicly available CHARLS data, with official authorization from the CHARLS project team for data usage.

### Assessment of sleep duration and quality

Data on sleep duration and quality were gathered through participants’ self-reported answers to these questions: “During the past month, how many hours of actual sleep did you get at night (average hours for one night)?” and “During the last week, my sleep was restless.” With reference to the prevalent nocturnal sleep duration threshold for seniors utilized in previous research [26–27], participants were categorized into short sleep (≤6 hours) and non-short sleep (>6 hours) groups based on baseline self-reported sleep duration. Our study focused on the decline in sleep duration, which was measured as the change in sleep duration from baseline (2015) to follow-up (2018). Declines were further classified into three categories: 1) reduction in nocturnal sleep of ≥1 h; 2) reduction in nocturnal sleep of ≥1.5 h; 3) reduction in nocturnal sleep of ≥2 h. We defined an increase in restless sleep days from baseline (2015) to follow-up (2018) as a deterioration in sleep quality.

### Assessment of air pollution

Air pollutants’ concentrations were acquired from the China High Air Pollutants (CHAP) database, offering high-resolution data about air pollutants on daily, monthly or annual scales over the long term. The platform employs a Space-Time Extra-Trees (STET) model, fusing ground measurements, satellite observations, and model simulation by artificial intelligence [28]. The STET model incorporates spatiotemporal information and displays good prediction accuracy. The cross-validated R-squared values (CV-R²) for PM_2.5_, PM_10_, NO_2_, SO_2,_ and CO stood at 89% with a root-mean-square error (RMSE) of 10.33 μg/m^3^, 89% with 15.77 μg/m^3^, 84% with 7.99 μg/m^3^, 84% with 10.7 μg/m^3^, and 80% with 0.29 μg/m^3^, respectively [29–31]. We examined air pollution exposure across 1- to 2-year periods before the two interviews to ascertain the prolonged impact of pollutants. Due to privacy restrictions, the participants’ detailed residential addresses were unavailable. Therefore, exposure levels from 124 cities were assessed at the municipal level and linked with participants’ information based on CHARLS city codes.

### Covariates

After considering past research, we adjusted for possible confounding variables within our analysis. Demographic covariates included residence (rural or urban), gender (male or female), age (45−60 years or ≥60 years), education (“uneducated”, “elementary school and below”, or “middle school and above”), and marital status (“married and living, cohabitating” or “divorced, married but separated, widowed, never married”). Health behavior variables included nap duration, smoking status (non-smoker or smoker), drinking status (non-drinker or drinker), and BMI. Variables pertinent to health conditions were also incorporated, including depressive symptoms and self-reported number of chronic diseases (0, 1, or ≥2). The latter included arthritis or rheumatism, kidney disease, chronic lung diseases, dyslipidemia, emotional or psychiatric problems, stomach or other digestive disease, hypertension, liver disease, heart attack, memory-related disease such as dementia, cancer or malignant tumor, diabetes or high blood sugar, stroke, and asthma. The 10-item Center for Epidemiologic Studies-Depression (CESD-10) scale was employed to evaluate depressive symptoms. A higher score implies greater severity of depression. Finally, analyses were conducted across regional categories (“Eastern”, “Central” and “Western”), taking into consideration variations in economic development and geographical location.

### Statistical analysis

Baseline features of individuals were examined through descriptive analysis. Continuous data were shown as mean ± SD, while categorical data were reported as counts and percentages. The absence rates for all covariates were below 20%. To maximize statistical power and reduce potential bias introduced by missing data, the random forest method was employed for imputation [32].

This study explored the association between inter-interview differences in air pollution exposure and changes in both sleep duration and quality. Δ Exposure to air pollution = the average concentrations over 12- or 24-month intervals before the follow-up in 2018 – the average concentrations over 12- or 24-month intervals before the baseline in 2015. Longitudinal analyses implemented the generalized linear mixed model (GLMM) with logit links to quantify the associations between varying air pollution exposure levels and sleep. Considering the potential regional impact, we took the pollutant concentration differences as the fixed-effect terms while designating the study region as the random-effect term. Due to collinearity among the differences in pollutant concentrations, each was analyzed in a separate model. We assessed the link between pollution levels and sleep by reporting outcomes per 10 μg/m^3^ increment for four pollutants. Given the substantially lower concentration levels of ambient CO exposure, results for CO were presented per 1 μg/m^3^ increase.

In order to mitigate the influence of confounding factors, three models were formulated:

Model 1 (Crude): Incorporated the pollutant concentration differences as the fixed-effect terms with study region as the random-effect term.

Model 2 (Demographic-adjusted): Adjusted for baseline marital status, education, residence, gender, and age.

Model 3 (Fully adjusted): Further adjusted for smoking status, drinking status, self-reported number of chronic diseases, depressive symptoms, nap duration, and BMI at baseline.

The dose-response relationship between pollutant exposure differences and studied outcomes was explored using the restricted cubic spline (RCS) regression model. After adjustment for all covariates, interaction analyses were performed for 1-year pollutant exposure difference to assess potential effect modification by age, sex, residence, regional category, and chronic disease status. Stratified analyses were subsequently performed to verify the interaction findings. Several sensitivity assessments were done to evaluate robustness. (1) To examine how varying exposure periods impact outcomes, we changed pollutant exposure from 1 year to 2 years. (2) Treating the reduction in nighttime sleep (i.e., ≥ 1 h, 1.5 h, and 2 h) as an ordinal dependent variable, we further utilized the ordinal logistic regression model to verify the stability of the association between air pollution and reduced sleep duration. (3) Multivariable analyses were performed separately for sleep duration and quality, with adjustment for confounding covariates. We processed and analyzed the data using Stata 18.0 software.

## Results

### Baseline characteristics

This research involved 14856 individuals who completed interviews in both 2015 and 2018, after excluding those under 45 years old and those lacking complete sleep information. Among the participants, 7849 (52.8%) were aged 45−60 years, and 7007 (47.2%) were aged ≥60 years. 7195 (48.4%) were men, and 7661 (51.6%) were women. Geographically, the participants were distributed as follows: 5059 (34.1%) from the eastern region, 4850 (32.6%) from the central region, and 4947 (33.3%) from the western region. The CESD-10 score was 7.9 ± 6.3, and BMI was 24.1 ± 4.0. The mean nap duration was 0.6 ± 0.7 hours. Most participants resided in rural regions (62.0%), had received education (76.9%), were married or cohabitating (88.8%), were non-smokers (71.3%), were non-drinkers (63.7%), and had chronic diseases (81.9%). A total of 7742 (52.1%) reported experiencing restless sleep <1 day/week.

Of the participants, 7517 (50.6%) reported nighttime sleep duration ≤6 h, while 7339 (49.4%) reported nighttime sleep duration >6 hours. Significant differences were observed among groups with different sleep durations. Compared with those sleeping >6 hours, short sleepers (≤6 hours) were significantly older, more likely to be female, divorced, afflicted with chronic diseases, and without formal education. They also exhibited shorter nap durations, higher CESD-10 scores and poorer sleep quality. No significant differences in residence, smoking status, and drinking status were observed across groups.

Table 1 shows the annual average levels of air pollutants before the baseline. The average concentrations of PM_2.5_, PM_10_ and NO_2_ were 51.2 μg/m^3^, 88.3 μg/m^3^, and 28.4 μg/m^3^, respectively, exceeding the limits recommended by the World Health Organization (WHO) global air quality guidelines [33].

**Table 1:** Baseline characteristics stratified by sleep duration groups.

Characteristics	Overall (n = 14856)	Short sleep (n = 7517)	Non-short sleep (n = 7339)	*P*
Age, n (%)				<0.001
45-60	7849 (52.8)	3772 (50.2)	4077 (55.6)	
≥60	7007 (47.2)	3745 (49.8)	3262 (44.4)	
Gender, n (%)				<0.001
Male	7195 (48.4)	3436 (45.7)	3759 (51.2)	
Female	7661 (51.6)	4081 (54.3)	3580 (48.8)	
Residence, n (%)				0.174
Rural	9216 (62.0)	4623 (61.5)	4593 (62.6)	
Urban	5640 (38.0)	2894 (38.5)	2746 (37.4)	
Regional categories				<0.001
Eastern	5059 (34.1)	2356 (31.3)	2703 (36.8)	
Central	4850 (32.6)	2542 (33.8)	2308 (31.4)	
Western	4947 (33.3)	2619 (34.8)	2328 (31.7)	
Education, n (%)				0.034
Uneducated	3428 (23.1)	1787 (23.8)	1641 (22.4)	
Elementary school and below	6715 (45.2)	3409 (45.4)	3306 (45.0)	
Middle school and above	4713 (31.7)	2321 (30.9)	2392 (32.6)	
Marital status, n (%)				<0.001
Married and living/cohabitating	13195 (88.8)	6565 (87.3)	6630 (90.3)	
Divorced/married but separated/widowed/never married	1661 (11.2)	952 (12.7)	709 (9.7)	
Smoking status, n (%)				0.146
Non-smoker	10597 (71.3)	5402 (71.9)	5195(70.8)	
Smoker	4259 (28.7)	2115 (28.1)	2144 (29.2)	
Drinking status, n (%)				0.130
Non-drinker	9458 (63.7)	4830 (64.3)	4628 (63.1)	
Drinker	5398 (36.3)	2687 (35.7)	2711 (36.9)	
Depressive symptom (CESD-10 score, mean ± SD)	7.9 ± 6.3	9.2 ± 6.7	6.5 ± 5.6	<0.001
BMI, mean ± SD, kg/m^2^	24.1 ± 4.0	23.9 ± 4.1	24.2 ± 4.0	<0.001
Number of chronic diseases, n (%)				<0.001
0	2683 (18.1)	1180 (15.7)	1503 (20.5)	
1	3695 (24.9)	1764 (23.5)	1931 (26.3)	
≥2	8478 (57.1)	4573 (60.8)	3905 (53.2)	
Nap duration, mean ± SD, h	0.6 ± 0.7	0.6 ± 0.7	0.7 ± 0.8	<0.001
Restless sleep, n (%)				<0.001
<1 day/week	7742 (52.1)	2782 (37.0)	4960 (67.6)	
1-2 days/week	2077 (14.0)	1073 (14.3)	1004 (13.7)	
3-4 days/week	2094 (14.1)	1331 (17.7)	763 (10.4)	
5-7 days/week	2943 (19.8)	2331 (31.0)	612 (8.3)	
Pollutant concentration, μg/m^3^				
PM_2.5_	51.2 ± 16.2	50.9 ± 16.0	51.4 ± 16.5	0.166
PM_10_	88.3 ± 29.8	87.3 ± 29.5	89.3 ± 30.1	<0.001
NO_2_	28.4 ± 8.8	28.0 ± 8.7	28.8 ± 9.0	<0.001
SO_2_	29.3 ± 13.5	28.6 ± 13.2	29.9 ± 13.8	<0.001
CO	1.1 ± 0.3	1.1 ± 0.3	1.1 ± 0.3	0.035

Abbreviations: “Uneducated” means illiterate; “elementary school and below” includes those who have not graduated from elementary school, graduated from elementary school, or graduated from a home school; “middle school and above” includes those with a doctoral degree, master’s degree, bachelor’s degree, associate degree, vocational school, high school or middle school.

### Descriptive analysis of changes in participants’ sleep duration and quality

From 2015 to 2018, 38.6%, 22.6%, and 20.3% of participants reported a reduction in nighttime sleep of at least 1 h, 1.5 h, and 2 h, respectively, while 29.2% reported deteriorated sleep quality (Table 2).

**Table 2:** Descriptive analysis of participants’ sleep changes, n (%).

Categories	No	Yes
Decrease in nighttime sleep (≥1 h)	9128 (61.4)	5728 (38.6)
Decrease in nighttime sleep (≥1.5 h)	11495 (77.4)	3361 (22.6)
Decrease in nighttime sleep (≥2 h)	11833 (79.7)	3023 (20.3)
Deterioration of sleep quality	10518 (70.8)	4338 (29.2)

### Effects of air pollution exposure on sleep duration

**Fig 2:**
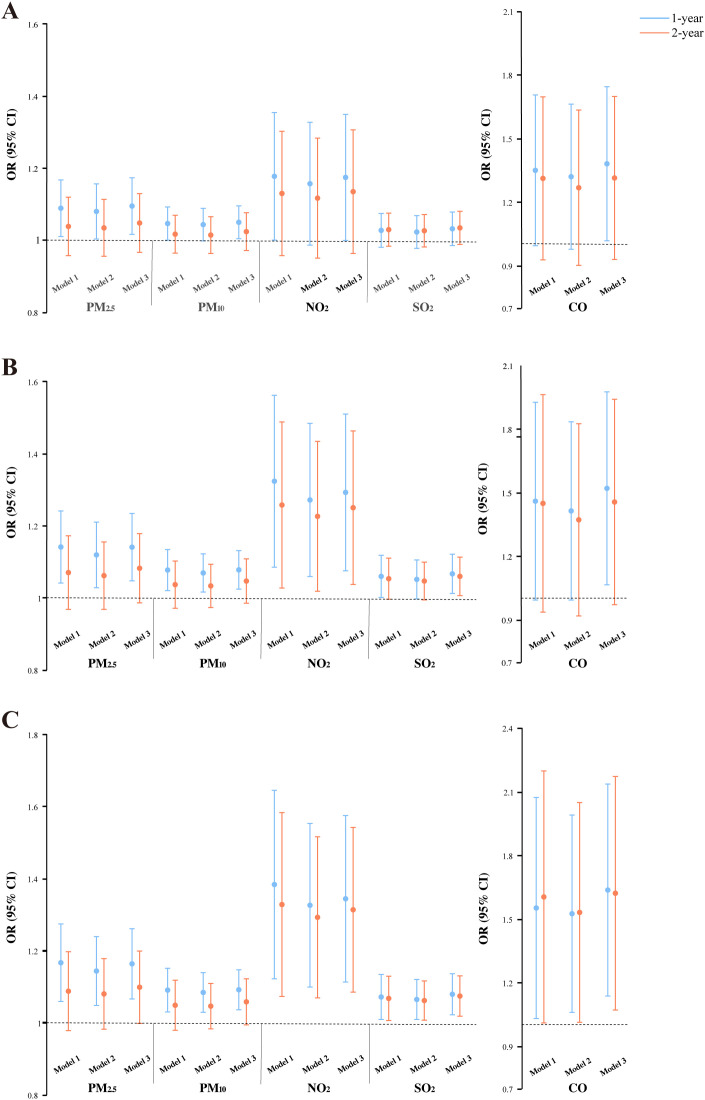
Associations between air pollution exposure and reductions in nighttime sleep of ≥1 h, 1.5 h, and 2 h. (A) A reduction in nighttime sleep of ≥1 h; (B) a reduction in nighttime sleep of ≥1.5 h; (C) a reduction in nighttime sleep of ≥2 **h.** Effects of PM_2.5_, PM_10_, NO_2_, and SO_2_ were calculated for a 10 μg/m^3^ increase in the average concentration difference over 1- or 2-year periods, while CO was calculated per 1 μg/m^3^ increase.

We estimated the impact of prolonged changes in exposure to each pollutant on nocturnal sleep duration. Model 3 (full adjustment) showed that all 1-year exposure differences positively correlated with reductions in sleep duration of both ≥1.5 h and ≥2 h (Figs 2B and 2C). Notably, CO exposure demonstrated the highest risk for a ≥ 1.5 h reduction (OR = 1.451; 95% CI: 1.065–1.975) and a ≥ 2 h reduction (OR = 1.557; 95% CI: 1.135–2.135) per 1 μg/m^3^ increment. For 2-year exposure differences, significant associations were identified for NO_2_ and SO_2_ with a ≥ 1.5 h reduction (Fig 2B), and for NO_2_, SO_2_, and CO with a ≥ 2 h reduction (Fig 2C). Interestingly, a reduction in nighttime sleep of ≥1 h was exclusively associated with increased 1-year exposure to PM_2.5_, PM_10_, and CO (Fig 2A). Moreover, the pollutants exhibited similar tendencies to reduce sleep duration across different exposure windows. The 1-year exposure difference showed stronger associations than the 2-year exposure difference did.

### Effects of air pollution exposure on sleep quality

**Fig 3:**
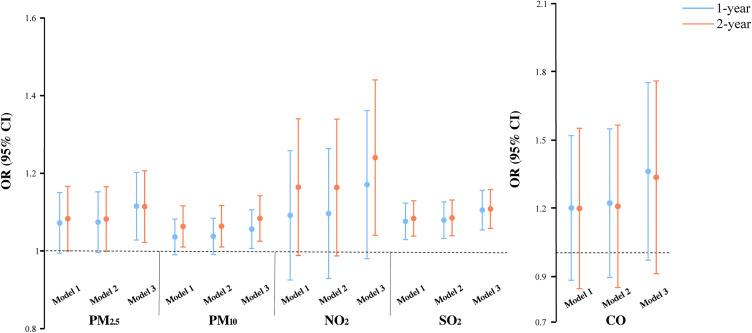
Associations between air pollution exposure and deteriorated sleep quality. Effects of PM_2.5_, PM_10_, NO_2_, and SO_2_ were calculated for a 10 μg/m^3^ increase in the average concentration difference over 1- or 2-year periods, while CO was calculated per 1 μg/m^3^ increase.

We estimated the impact of prolonged changes in exposure to each pollutant on sleep quality. In the fully adjusted model 3, increased exposure to PM_2.5_, PM_10,_ NO_2_ and SO_2_ was associated with an elevated risk of deteriorated sleep quality (Fig 3). Notably, 2-year exposure to NO_2_ demonstrated the highest risk, with a 22.3% higher risk per 10 μg/m^3^ increment (95% CI: 1.039–1.439). The effects of pollutants on sleep quality were similar across different exposure windows. The 2-year exposure difference consistently demonstrated stronger correlations than the 1-year exposure difference.

### Subgroup and interaction analysis

**Fig 4:**
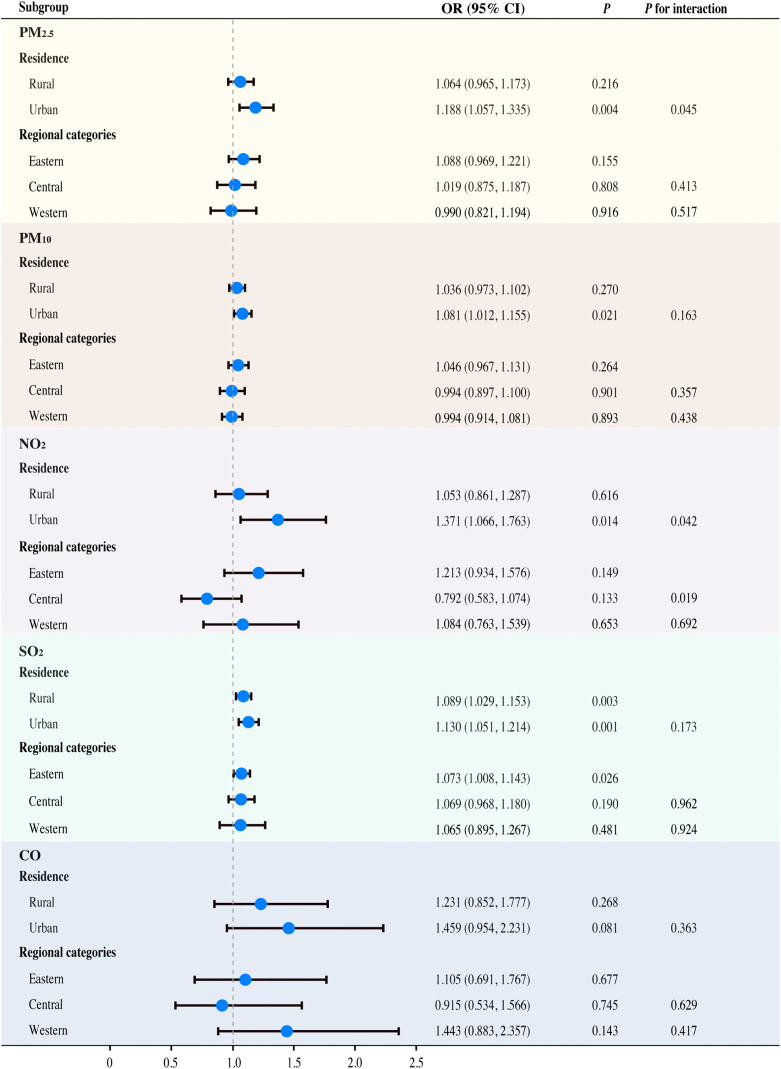
Interaction of residence and regional category on sleep quality. The effects of PM_2.5_, PM_10_, NO_2_, and SO_2_ were calculated for a 10 μg/m^3^ increase in the average concentration difference over 1- or 2-year periods, while CO was calculated per 1 μg/m^3^ increase.

In subgroup analyses, we examined the associations between 1-year exposure differences to air pollutants and sleep outcomes, stratified by age, sex, residence, regional category, and chronic disease status. As shown in Fig 4, residence significantly modified the associations of PM_2.5_ (*P* for interaction = 0.045) and NO_2_ (*P* for interaction = 0.042) with sleep quality, with urban residents showing stronger associations than rural residents. The analysis also indicated that regional categories modified the association between NO_2_ and sleep quality, although no significant association was found within the individual Eastern or Central subgroups. No significant effect modification was found for age, sex, or chronic disease status on sleep quality (S2 Table). Given the lack of correction for multiple testing, these interaction findings should be interpreted as exploratory. Results from simple stratified analyses were consistent with those from the interaction analyses. In contrast, none of these factors modified the associations with sleep duration (S1 Table).

### Dose-response relationship analysis

**Fig 5:**
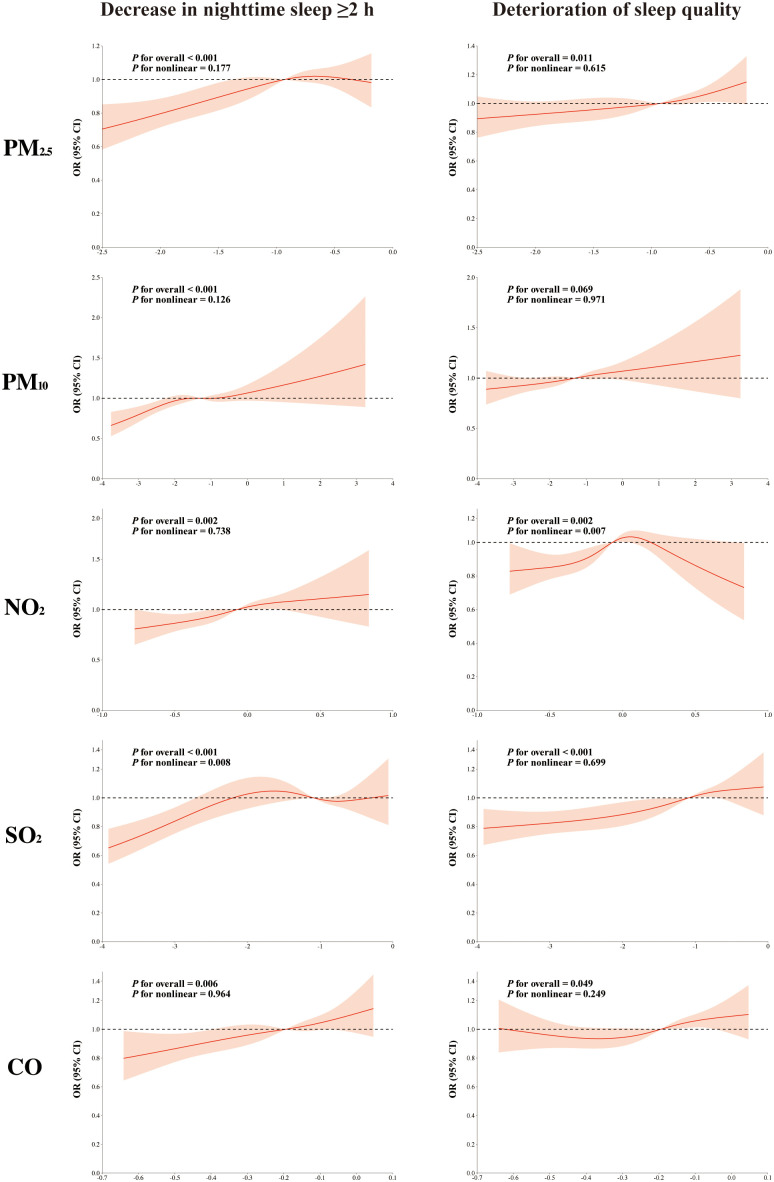
Analysis of restricted cubic spline regression. The effects of PM_2.5_, PM_10_, NO_2_, and SO_2_ were calculated for a 10 μg/m^3^ increase in the average concentration difference over the 1-year period, while CO was calculated per 1 μg/m^3^ increase.

To investigate the dose-response relationship between pollutant exposure differences and studied outcomes, we performed an analysis using the RCS model. After fully adjusting for confounders, significant linear associations were observed for the exposure differences of PM_2.5_, PM_10_, NO_2_, and CO with a decrease in nighttime sleep of ≥2 h (*P* for nonlinear = 0.177, 0.126, 0.738, and 0.964, respectively). Furthermore, the exposure differences of PM_2.5_, SO_2_, and CO showed linear associations with deteriorated sleep quality (*P* for nonlinearity = 0.615, 0.699, and 0.249, respectively). With increasing exposure, the RCS curves showed an increased risk of both reduced sleep duration and deteriorated sleep quality (Fig 5).

### Sensitivity Analysis

In the sensitivity analysis, we observed that the aforementioned associations persisted when the exposure window was extended from 1 to 2 years (Figs 2 and 3). Table 3 reports the ordinal logistic regression results of 1-year exposure differences to pollutants on reduced sleep duration, revealing significant associations between increased exposure and reduced sleep duration for all pollutants, which align with the GLMM results, suggesting that the model did not influence our findings. Finally, associations between air pollution exposure and sleep remained robust after excluding 3543 participants with unaltered sleep duration and 7264 participants with unaltered sleep quality during the follow-up period, respectively (S3 Table). The consistency across various sensitivity analyses demonstrates the robustness of the primary findings.

**Table 3:** Ordinal logistic regression results for reduced sleep duration.

Categories	OR	95% CI		*P*
PM_2.5_	1.107	1.046	1.172	<0.001
PM_10_	1.059	1.022	1.096	0.001
NO_2_	1.204	1.069	1.356	0.002
SO_2_	1.042	1.007	1.079	0.020
CO	1.417	1.146	1.751	0.001

Note: The effects of PM_2.5_, PM_10_, NO_2_, and SO_2_ were calculated for a 10 μg/m^3^ increase in the average concentration difference over the 1-year period, while CO was calculated per 1 μg/m^3^ increase.

## Discussion

This study provides novel evidence from a nationwide prospective cohort on the associations between changes in long-term air pollution exposure and sleep outcomes in middle-aged and older populations. We discovered that higher exposure to PM_2.5_, PM_10_, NO_2_, SO_2_, and CO was significantly correlated with diminished sleep duration in middle-aged and older populations. Interestingly, this effect was more pronounced with a ≥ 2-hour sleep loss. Second, higher PM_2.5_, PM_10_, NO_2_, and SO_2_ exposure was significantly linked to deteriorated sleep quality, with NO_2_ exposure exhibiting the highest risk. Different exposure windows exhibited distinct impacts on sleep duration and quality, with 1-year exposure difference exerting a more significant influence on sleep duration and 2-year exposure difference demonstrating a more substantial effect on sleep quality. Furthermore, we noted that air pollution exposure significantly impacted sleep quality in urban residents.

Several investigations have confirmed the negative impact of prolonged air pollution exposure on sleep health by disrupting sleep rhythms and structure through distinct physiological mechanisms [34–35]. Nevertheless, limited research has explicitly examined the correlation between long-term changes in air pollution and sleep duration. Our research indicated that higher PM_2.5_, PM_10_, NO_2_, SO_2_, and CO exposure significantly reduced sleep duration among middle-aged and older populations. Prior research has reported associations between air pollution and sleep duration, but their findings are inconsistent. Variations in types of pollutants, study populations, study designs, and methods of sleep measurement may lead to such discrepancies, with the majority of prior studies relying on cross-sectional designs. Our findings align with those from a large-scale cross-sectional study utilizing data from the UK Biobank, comprising 5,976 cases and 97,160 controls [36]. The study identified ambient particulate matter as a threat to sleep health, contributing to issues like obstructive sleep apnea (OSA) and insomnia. A cohort study involving 16,889 first-year university students in Beijing, China, discovered that the 7-day average concentrations of NO_2_, PM_2.5_, and PM_10_ showed significant associations with subjectively reported reductions in nighttime sleep [37]. Nevertheless, the reliability of these findings may be constrained by the brief exposure duration. Another Chinese prospective investigation found that sustained exposure (for 3 years) to elevated PM_2.5_ and PM_10_ concentrations may diminish nocturnal sleep duration in middle-aged and older populations [38]. Nonetheless, the designs and populations of these studies diverged from our own. This study quantified how changes in air pollution exposure across interviews drive sleep duration and quality declines. The longitudinal design with a difference-in-differences approach strengthens the temporal plausibility of the observed associations compared to cross-sectional studies [39–40], although residual confounding cannot be entirely ruled out. Conversely, a team from Peking University found that, according to data from wearable devices, prolonged exposure to various air pollutants was negatively correlated with deep sleep duration, while being positively correlated with total sleep duration [20]. A small-sample cohort study in the United States objectively recorded via actigraphy that elevated short-term (1-day) and medium-term (60-day) PM_2.5_ exposure was associated with greater variability in nocturnal sleep duration among adolescents in Central Pennsylvania, although no effect on sleep duration itself was observed [41]. This discrepancy may stem from variations in objectively measured and self-reported sleep duration.

The second finding of this study was that higher exposure to PM_2.5_, PM_10_, NO_2_ and SO_2_ was correlated with deteriorated sleep quality among middle-aged and older populations. Despite high heterogeneity in study populations, pollutant types, and sleep measurement methods, which impede direct comparison of existing evidence, there is broad recognition that air pollution exposure compromises sleep quality [42–44]. For instance, a Mexican pregnancy cohort study involving 397 maternal-infant pairs revealed a correlation between early gestational PM_2.5_ exposure and reduced sleep efficiency in preschool-aged children [42]. A cohort study by researchers from Wuhan University revealed that prolonged exposure to air pollutants, including NO_2_, PM_2.5_, and PM_10_, was significantly associated with poor sleep quality among individuals living in rural China [43]. Air pollution can compromise sleep quality by exacerbating OSA [44]. Wang et al. observed that chronic NO_2_ exposure correlated with higher Pittsburgh Sleep Quality Index (PSQI) scores among the elderly in rural China [45].

Prior studies on air pollution and sleep predominantly concentrated on ambient particulate matter. PM_2.5_ and PM_10_ concentrations exceed national standards and necessitate stringent regulation through rigorous enforcement of current protective measures. Our study innovatively found that SO_2_ and CO negatively impact sleep, even when levels remain under the thresholds established by the WHO global air quality guidelines. This discovery underscores the necessity of raising public awareness about pollutants’ sleep-disrupting effects and highlights their profound public health implications.

The mechanisms linking air pollution to sleep disturbances, though not yet fully elucidated, are understood to involve multiple biological pathways. First, air pollutant-induced respiratory tract irritation triggers upper airway mucosal inflammation and edema [46–47]. This increases the likelihood of apnea and hypoxia, consequently disrupting sleep. Moreover, air pollutants compromise the blood-brain barrier (BBB) [48], leading to heightened neuroinflammation and changes in brain neurotransmitter levels. Certain pollutants can also infiltrate the CNS directly through the olfactory nerve, bypassing the BBB and impairing neurological functions [49]. Moreover, sunshine prompts the pineal gland to release melatonin, a hormone essential for regulating circadian rhythms and facilitating the initiation and maintenance of sleep [50]. However, ambient particulate matter causes severe haze, which not only reduces sunlight penetration but may also decrease individuals’ motivation for outdoor sun exposure [51], thereby disrupting melatonin secretion.

A more pronounced effect of air pollution on sleep quality was noted among urban residents than among those residing in rural regions. A study showed that the risk of insomnia among rural residents was 16% lower than that of urban residents [52]. The possible reason is that urban residents typically face more complex exposure to air pollution, encompassing traffic emissions (NO_2_, PM_2.5_, polycyclic aromatic hydrocarbons), industrial discharges (SO_2_, heavy metals), and secondary pollutants. Moreover, the extensive vegetation in rural areas mitigates air pollution through particulate matter capture and airborne contaminant uptake [53]. Thus, air pollution inflicts more severe harm on urban residents.

### Limitations

Several limitations of this study warrant critical discussion. First, the reliance on self-reported sleep data introduces the potential for recall bias and non-differential misclassification. Participants may systematically inaccurately recall their sleep patterns, which could bias the observed associations, likely attenuating the effect estimates towards the null. Second, exposure assessment at the municipal level, necessitated by the unavailability of precise residential addresses, constitutes a significant limitation. That is to say, the participants within the same municipality were assigned the same exposure level to all participants, which may not reflect individual-level exposure due to micro-environmental variations. Third, our analysis could not account for residential mobility during the study period. Participants who moved between cities with differing air pollution levels may have been misclassified, further contributing to exposure measurement error. Fourth, despite adjusting for a wide range of covariates, the potential for residual confounding persists. Important sleep-related factors such as caffeine or tea consumption, nighttime light exposure, and ambient noise were not measured in the CHARLS dataset and thus could not be adjusted for. Notwithstanding these limitations, our study exhibits notable strengths, including a prospective difference-in-differences design and the simultaneous investigation of five air pollutants. While this national study provides valuable evidence, it needs to be interpreted with caution due to its limitations. Future work with individual-level exposure data, residential tracking, and expanded confounding factors is needed to enhance causal relationships.

## Conclusion

In summary, this study provides evidence that long-term exposure to several air pollutants is associated with adverse sleep outcomes in Chinese adults aged 45 years and above. Especially among urban residents, the adverse effects on sleep quality are more pronounced. From a public health perspective, these findings highlight the potential benefits of improving air quality and warrant further investigation into the underlying mechanisms by which air pollution affects sleep.

## Supporting information

S1 Table Interaction and simple stratified analyses on a decrease in nighttime sleep of ≥2 h. Note: The effects of PM_2.5_, PM_10_, NO_2_, and SO_2_ were calculated per 10 μg/m^3^ increase in the average concentration difference over the 1-year period, while CO was calculated per 1 μg/m^3^ increase.
(DOCX)

S2 Table Interaction and simple stratified analyses on sleep quality. Note: The effects of PM_2.5_, PM_10_, NO_2_, and SO_2_ were calculated per 10 μg/m^3^ increase in the average concentration difference over the 1-year period, while CO was calculated per 1 μg/m^3^ increase.
(DOCX)

S3 Table Associations between exposure to air pollution and sleep duration/quality after excluding samples with no change in sleep duration and sleep quality, respectively. Note: (a) excluding samples with no change in sleep duration; (b) excluding samples with no change in sleep quality. The effects of PM_2.5_, PM_10_, NO_2_, and SO_2_ were calculated per 10 μg/m^3^ increase in the average concentration difference over 1- or 2-year periods, while CO was calculated per 1 μg/m^3^ increase.
(DOCX)
