# Supplementary material for: Association of long-term exposure to air pollution with sleep among middle-aged and older adults in China: A nationwide study from 2015 to 2018
Source: PLoS One. 2026 Mar 27;21(3):e0336665. doi: 10.1371/journal.pone.0336665 (PMC13028504; doi:10.1371/journal.pone.0336665)
Supplement: S1 Table — Note: The effects of PM2.5, PM10, NO2, and SO2 were calculated per 10 μg/m3 increase in the average concentration difference over the 1-year period, while CO was calculated per 1 μg/m3 increase. (DOCX) [file pone.0336665.s001.docx]

| Characteristics | Subgroup | PM_2.5_ | | PM_10_ | | NO_2_ | | SO_2_ | | CO | |
| --- | --- | --- | --- | --- | --- | --- | --- | --- | --- | --- | --- |
|  |  | OR (95%CI) | *P* | OR (95%CI) | *P* | OR (95%CI) | *P* | OR (95%CI) | *P* | OR (95%CI) | *P* |
| Age | 45-60 | 1.161 (1.032, 1.306) | 0.997 | 1.093 (1.019, 1.173) | 0.915 | 1.337 (1.047, 1.707) | 0.875 | 1.073 (0.997, 1.156) | 0.976 | 1.552 (0.996, 2.418) | 0.812 |
|  | ≥60 | 1.168 (1.034, 1.319) |  | 1.093 (1.015, 1.177) |  | 1.313 (1.024, 1.685) |  | 1.083 (1.005, 1.169) |  | 1.514 (0.961, 2.384) |  |
| Gender | Male | 1.146 (1.023, 1.283) | 0.981 | 1.090 (1.017, 1.168) | 0.839 | 1.170 (0.922, 1.485) | 0.235 | 1.046 (0.974, 1.123) | 0.427 | 1.454 (0.954, 2.215) | 0.902 |
|  | Female | 1.169 (1.054, 1.296) |  | 1.090 (1.023, 1.160) |  | 1.456 (1.184, 1.791) |  | 1.107 (1.040, 1.178) |  | 1.705 (1.162, 2.501) |  |
| Residence | Rural | 1.137 (1.027, 1.257) | 0.463 | 1.091 (1.023, 1.163) | 0.991 | 1.283 (1.048, 1.570) | 0.561 | 1.087 (1.022, 1.156) | 0.778 | 1.761 (1.213, 2.557) | 0.334 |
|  | Urban | 1.209 (1.058, 1.383) |  | 1.090 (1.010, 1.176) |  | 1.428 (1.063, 1.919) |  | 1.068 (0.976, 1.168) |  | 1.374 (0.818, 2.308) |  |
| 区域类别 | 1 | 1.127 (0.996, 1.274) |  | 1.063 (0.976, 1.157) |  | 1.128 (0.839, 1.515) |  | 1.064 (0.993, 1.141) |  | 1.340 (0.806, 2.227) |  |
|  | 2 | 1.154 (0.975, 1.365) | 0.999 | 1.083 (0.967, 1.213) | 0.959 | 1.438 (1.038, 1.991) | 0.387 | 1.057 (0.944, 1.183) | 0.768 | 1.499 (0.824, 2.726) | 0.904 |
|  | 3 | 1.009 (0.821, 1.239) | 0.238 | 1.048 (0.957, 1.148) | 0.614 | 1.036 (0.703, 1.527) | 0.583 | 0.919 (0.765, 1.105) | 0.133 | 1.389 (0.812, 2.377) | 0.880 |
| Number of chronic diseases | 0 | 1.078 (0.912, 1.273) |  | 1.051 (0.946, 1.168) |  | 1.318 (0.941, 1.846) |  | 1.032 (0.936, 1.137) |  | 1.386 (0.742, 2.589) |  |
|  | 1 | 1.161 (1.001, 1.347) | 0.454 | 1.070 (0.979, 1.171) | 0.989 | 1.192 (0.889, 1.597) | 0.707 | 1.119 (1.022, 1.225) | 0.196 | 1.610 (0.922, 2.812) | 0.616 |
|  | ≥2 | 1.191 (1.069, 1.327) | 0.332 | 1.112 (1.043, 1.185) | 0.592 | 1.392 (1.109, 1.746) | 0.799 | 1.081 (1.009, 1.159) | 0.433 | 1.673 (1.117, 2.505) | 0.531 |
