# Supplementary material for: Association of long-term exposure to air pollution with sleep among middle-aged and older adults in China: A nationwide study from 2015 to 2018
Source: PLoS One. 2026 Mar 27;21(3):e0336665. doi: 10.1371/journal.pone.0336665 (PMC13028504; doi:10.1371/journal.pone.0336665)
Supplement: S2 Table — Note: The effects of PM2.5, PM10, NO2, and SO2 were calculated per 10 μg/m3 increase in the average concentration difference over the 1-year period, while CO was calculated per 1 μg/m3 increase. (DOCX) [file pone.0336665.s002.docx]

| Characteristics | Subgroup | PM_2.5_ | | PM_10_ | | NO_2_ | | SO_2_ | | CO | |
| --- | --- | --- | --- | --- | --- | --- | --- | --- | --- | --- | --- |
|  |  | OR (95%CI) | *P* | OR (95%CI) | *P* | OR (95%CI) | *P* | OR (95%CI) | *P* | OR (95%CI) | *P* |
| Age | 45-60 | 1.156 (1.059, 1.262) | 0.447 | 1.069 (1.013, 1.129) | 0.734 | 1.201 (0.995, 1.451) | 0.855 | 1.112 (1.055, 1.173) | 0.738 | 1.251 (0.889, 1.761) | 0.293 |
|  | ≥60 | 1.059 (0.951, 1.178) |  | 1.038 (0.972, 1.108) |  | 1.099 (0.881, 1.372) |  | 1.098 (1.030, 1.171) |  | 1.406 (0.946, 2.089) |  |
| Gender | Male | 1.053 (0.949, 1.169) | 0.440 | 1.029 (0.966, 1.097) | 0.549 | 1.000 (0.804, 1.245) | 0.146 | 1.078 (1.013, 1.148) | 0.805 | 1.463 (1.001, 2.138) | 0.160 |
|  | Female | 1.158 (1.053, 1.273) |  | 1.073 (1.013, 1.137) |  | 1.310 (1.076, 1.595) |  | 1.125 (1.062, 1.192) |  | 1.181 (0.819, 1.703) |  |
| Residence | Rural | 1.064 (0.965, 1.173) | **0.045** | 1.036 (0.973, 1.102) | 0.163 | 1.053 (0.861, 1.287) | **0.042** | 1.089 (1.029, 1.153) | 0.173 | 1.231 (0.852, 1.777) | 0.363 |
|  | Urban | 1.188 (1.057, 1.335) |  | 1.081 (1.012, 1.155) |  | 1.371 (1.066, 1.763) |  | 1.130 (1.051, 1.214) |  | 1.459 (0.954, 2.231) |  |
| 区域类别 | 1 | 1.088 (0.969, 1.221) |  | 1.046 (0.967, 1.131) |  | 1.213 (0.934, 1.576) |  | 1.073 (1.008, 1.143) |  | 1.105 (0.691, 1.767) |  |
|  | 2 | 1.019 (0.875, 1.187) | 0.413 | 0.994 (0.897, 1.100) | 0.357 | 0.792 (0.583, 1.074) | **0.019** | 1.069 (0.968, 1.180) | 0.962 | 0.915 (0.534, 1.566) | 0.629 |
|  | 3 | 0.990 (0.821, 1.194) | 0.517 | 0.994 (0.914, 1.081) | 0.438 | 1.084 (0.763, 1.539) | 0.692 | 1.065 (0.895, 1.267) | 0.924 | 1.443 (0.883, 2.357) | 0.417 |
| Number of chronic diseases | 0 | 1.223 (1.056, 1.416) |  | 1.146 (1.045, 1.257) |  | 1.333 (0.990, 1.796) |  | 1.126 (1.033, 1.229) |  | 1.087 (0.630, 1.873) |  |
|  | 1 | 1.050 (0.912, 1.209) | 0.259 | 1.012 (0.928, 1.102) | 0.119 | 1.011 (0.760, 1.345) | 0.264 | 1.054 (0.968, 1.148) | 0.481 | 1.187 (0.699, 2.017) | 0.613 |
|  | ≥2 | 1.113 (1.000, 1.238) | 0.241 | 1.052 (0.987, 1.122) | 0.126 | 1.173 (0.937, 1.469) | 0.494 | 1.121 (1.050, 1.197) | 0.832 | 1.451 (0.975, 2.160) | 0.516 |
