# Supplementary material for: Association of long-term exposure to air pollution with sleep among middle-aged and older adults in China: A nationwide study from 2015 to 2018
Source: PLoS One. 2026 Mar 27;21(3):e0336665. doi: 10.1371/journal.pone.0336665 (PMC13028504; doi:10.1371/journal.pone.0336665)
Supplement: S3 Table — Note: (a) excluding samples with no change in sleep duration; (b) excluding samples with no change in sleep quality. The effects of PM2.5, PM10, NO2, and SO2 were calculated per 10 μg/m3 increase in the average concentration difference over 1- or 2-year periods, while CO was calculated per 1 μg/m3 increase. (DOCX) [file pone.0336665.s003.docx]

| Categories | Exposure window | PM_2.5_ | | PM_10_ | | NO_2_ | | SO_2_ | | CO | |
| --- | --- | --- | --- | --- | --- | --- | --- | --- | --- | --- | --- |
|  |  | OR (95%CI) | *P* | OR (95%CI) | *P* | OR (95%CI) | *P* | OR (95%CI) | *P* | OR (95%CI) | *P* |
| Decrease in nighttime sleep (≥1 h) ^a^ | 1-year | 1.080 (0.995, 1.171) | 0.065 | 1.042 (0.991, 1.095) | 0.107 | 1.119 (0.943, 1.326) | 0.197 | 1.029 (0.978, 1.083) | 0.271 | 1.373 (1.014, 1.860) | 0.040 |
|  | 2-year | 1.034 (0.948, 1.129) | 0.450 | 1.014 (0.957, 1.074) | 0.635 | 1.073 (0.903, 1.274) | 0.423 | 1.031 (0.980, 1.084) | 0.235 | 1.289 (0.918, 1.809) | 0.143 |
| Decrease in nighttime sleep (≥1.5 h) ^a^ | 1-year | 1.126 (1.036, 1.224) | 0.005 | 1.072 (1.019, 1.127) | 0.008 | 1.239 (1.042, 1.474) | 0.015 | 1.065 (1.011, 1.122) | 0.018 | 1.455 (1.065, 1.987) | 0.019 |
|  | 2-year | 1.073 (0.980, 1.174) | 0.126 | 1.042 (0.982, 1.106) | 0.176 | 1.198 (1.005, 1.428) | 0.044 | 1.057 (1.004, 1.112) | 0.035 | 1.374 (0.968, 1.951) | 0.075 |
| Decrease in nighttime sleep(≥2 h) ^a^ | 1-year | 1.150 (1.056, 1.252) | 0.001 | 1.086 (1.031, 1.144) | 0.002 | 1.290 (1.081, 1.540) | 0.005 | 1.078 (1.022, 1.137) | 0.006 | 1.558 (1.133, 2.144) | 0.006 |
|  | 2-year | 1.090 (0.994, 1.196) | 0.068 | 1.054 (0.992, 1.121) | 0.091 | 1.263 (1.056, 1.510) | 0.011 | 1.071 (1.017, 1.129) | 0.010 | 1.525 (1.066, 2.181) | 0.021 |
| Deterioration of sleep quality ^b^ | 1-year | 1.209 (1.090, 1.341) | <0.001 | 1.093 (1.026, 1.165) | 0.006 | 1.266 (1.012, 1.584) | 0.039 | 1.140 (1.070, 1.214) | <0.001 | 1.466 (0.983, 2.185) | 0.060 |
|  | 2-year | 1.225 (1.098, 1.366) | <0.001 | 1.144 (1.065, 1.229) | <0.001 | 1.376 (1.104, 1.716) | 0.004 | 1.150 (1.083, 1.222) | <0.001 | 1.386 (0.889, 2.161) | 0.150 |
